# A Review on Planted (*l*, d) Motif Discovery Algorithms for Medical Diagnose

**DOI:** 10.3390/s22031204

**Published:** 2022-02-05

**Authors:** Satarupa Mohanty, Prasant Kumar Pattnaik, Ahmed Abdulhakim Al-Absi, Dae-Ki Kang

**Affiliations:** 1 School of Computer Engineering, KIIT Deemed to be University, Bhubaneswar 751024, India; satarupafcs@kiit.ac.in (S.M.); patnaikprasant@gmail.com (P.K.P.); 2 Department of Smart Computing, Kyungdong University, 46 4-gil, Bongpo, Gosung 24764, Korea; absiahmed@kduniv.ac.kr; 3 Department of Computer & Information Engineering, Dongseo University, 47 Jurye-ro, Sasang-gu, Busan 47011, Korea

**Keywords:** probabilistic approach, local search approach, evolutionary approach, search tree, suffix tree, mismatch tree, tries, hashing

## Abstract

Personalized diagnosis of chronic disease requires capturing the continual pattern across the biological sequence. This repeating pattern in medical science is called “Motif”. Motifs are the short, recurring patterns of biological sequences that are supposed signify some health disorder. They identify the binding sites for transcription factors that modulate and synchronize the gene expression. These motifs are important for the analysis and interpretation of various health issues like human disease, gene function, drug design, patient’s conditions, etc. Searching for these patterns is an important step in unraveling the mechanisms of gene expression properly diagnose and treat chronic disease. Thus, motif identification has a vital role in healthcare studies and attracts many researchers. Numerous approaches have been characterized for the motif discovery process. This article attempts to review and analyze fifty-four of the most frequently found motif discovery processes/algorithms from different approaches and summarizes the discussion with their strengths and weaknesses.

## 1. Introduction

Living organisms are composed of firmly analogous entities where isolation of a single entity is nonexistent. Organisms are composed of organs, organs of tissues, tissues of cells, and finally, cells are developed from molecules. Coordination for the amalgamation of living systems is monitored and achieved through multiple layers of interdependence: molecules transfer messages from cell to cell and organ to organ. The biological system is better studied by segment-wise or level wise, starting from the biology of the cell, because each level into which the biological system is fragmented are interconnected.

Motifs, in computational biology, are short, repeating patterns of biological sequences that are supposed to carry some biological signification. These motifs are important for the analysis and interpretation of various biological issues like drug design, human disease, gene function, etc. Searching for these patterns is essential to unravel the mechanisms of gene expression. Thus, motif identification possesses one of the vital roles in the studies of the biological disciplines and attracts many researchers. Numerous approaches have been characterized by different motif finding problems, and from these, the motif search problem formulated with substitutions, insertions, and deletions, such as in the planted (*l*, d) motif search, is particularly a challenge. The problem of planted (*l*, d) motif receives t biological sequences and integers *l* and d, with 0 ≤ d < *l* and outputs the length *l* biological sequences that occur in every input sequence with maximum d mismatches.

The major objectives of motif search in computational biology are the following:

1. Management, analysis, and interpretation of huge biological sequences using computational techniques from computer science and mathematics.
2. Development of innovative approaches and technologies for the diagnosis and understanding of the genetic diseases, for innovation and design of drugs for their medication, and for the improvement of healthcare.

This article reviews, analyzes, and compares various algorithms incorporated in the past decade by the researchers towards the motif search in the biological domain. This study opens the door for the researchers especially working in the domain of algorithmic-based solutions for finding motif. This paper is organized into nine sections. The paper starts by discussing the computational approach to bioinformatics with its related issues in Section 3 and Section 2, respectively. Then, it throws light upon the transcription factor binding site motif discovery problem in Section 4 and its related stumbling block in the search process in Section 5. Section 6 classifies the motif finding problem, and Section 7 delineates the planted (*l*, d) motif discovery problem briefly. Section 8 is the essence of the paper which reviews the groundwork done in the domain of the planted (*l*, d) motif search problem from every aspect. Finally, the paper concludes in Section 9.

## 2. Computational Approach to Bioinformatics

The mathematical modelling of computational approaches to bioinformatics uses numerical model predictions, simulation, and analysis. As a result, it produces a voluminous size of biological data which require a revolutionary technique for analyzing, processing, and archiving [1]. As a consequence, computational molecular biology is treated as an interdisciplinary domain that builds upon the fields of computer science, biology, chemistry, physics, mathematics, statistics, and engineering. Our understanding of the progress of the biological system is based upon its computational approach and technique. Owing to its requirements and demands, it is requisite to develop methods and models to understand the complexities of numerous biological networks and biomolecules at the system level. This approach opens the door to the understanding and examination of the ever-changing interrelationships among the components, their regulatory patterns, their impact on each other, and so on. The challenges of computational bioinformatics are to furnish the approaches to reconstructing the high-throughput divergent data sets into biological intuitions employing the underlying systems. There exist numerous computational advances to address biological problems, yet they leave enough room for enhancement.

## 3. Issues in Bioinformatics Problems

In the domain of bioinformatics, a variety of challenges are present. Among these, the critical issue lies in the organization and representation of the voluminous biological data properly through the DNA sequencing method keeping a balance with its continual growth. One more important issue is to draw out the detail of competent and knowledgeable data from the huge volume of the repository and thereby facilitate the advance insights into the life system. However, in bioinformatics, interesting problems and their implementation, such as motif search, inference of perfect phylogenies, and multiple optimal sequence alignment, are NP-complete by nature, and also computationally, these are extremely rigorous. Hence, in bioinformatics, high-performance computing characterizes the demanding challenge for bioinformatics as well as for the researcher owing to its strong interdisciplinary features from high-performance computing, theoretical computer science, and biology. These features are to be blended into an exclusive program where the techniques of concurrent pipelining and parallel simulation will be used to reduce the computation time.

## 4. The Transcription Factor Binding Site Motif Discovery Problem

The genetic information of organisms is carried out by the deoxyribonucleic acid chain, DNA, from the parent cell to its child cell. This genetic information is put into code over a group of four specific nucleotides, Adenine, Thymine Guanine, and Cytosine, which can be abbreviated as A, T, G, C, respectively. The short segments present in the long DNA sequence are designated as genes whose function is to produce proteins. For living organisms, proteins are the most important part that plays a vital character in defining cell structure and cell function. In regards to the impact on gene expression, a molecule named Transcription Factor (TF) binds a particular section of a gene called Transcription Factor Binding Site (TFBS). In the process of decoding, the individual Transcription Factor is capable of binding several TFBS of different genes. These Transcription Factors explicitly are called the motif.

Motifs in the biological domain are the short and recurring patterns of amino acids or nucleotides possessing some biological significance. They particularly regulate the functional and evolutionary relationships. These can start off or shut off the transcription process and are responsible for regulating gene expression. The motif appears randomly in the gene regulatory region of either one gene or several genes. It characterizes an important part in the recognition of genes, getting an insight into their regulation process and leading to several solutions in computational biology. For instance, motifs are used in PCR primer design, genetic probe design, potential drug targets and their design, production of diagnostic probes, discovery of protein families with an unbiased consensus, etc. Additionally, motif search can be used for discovering regulatory details within biological sequences, discovering binding sites in amino acids, searching for protein domains, and splicing information [2].

## 5. Issues with Motif Discovery Problems

In the domain of computational bioinformatics, motif identification is the most crucial issue. The localization and characterization of motifs are the essential factors in gaining fundamental knowledge of the structure of a gene and understanding evolutionary as well as functional relationships, and are proven to be a vital context in the domain of microenvironment. However, challenging issues make the search process difficult.

The motif search process experiences the following challenges [3]:

1. The search process begins without the knowledge of the location and structure of the motif.
2. There is no perfect matching between the motif and the real conserved sequence as the motif is implanted approximately in the biological sequences.
3. The motif length compared to the regulatory regions which hold the motif is very short, and above that mutation of the true motif, it becomes more complicated.
4. In some cases, motif binding sites and the coding region are found to be far away from each other, which leads to confusion in deciding the portion of the DNA sequence to be analyzed.
5. In some cases, the binding sites are present in just the opposite strand from their operated coding sequence.
6. The problem of motif search is itself the NP-Complete problem and is not solvable in polynomial-time.

In spite of the challenging aspects of the motif search problem, some of its dimensions make it worthwhile. In regard to the essence of benefits incurred from the problem of motif identification, there is scope for considerable research in this domain. Researchers have enhanced the motif search algorithm and tools very promisingly and progress is cause for optimim, even though the absence of a specific method which can assemble all relevant elements is still a matter for consideration. In the past, the motif was determined experimentally through a few motif finding tools such as gel_shift [4], DNA Footprinting [5], and SELEX [6] which can approximate or determine the weak motif only. However, scrutinizing the best motif finding tool out of several tools based on their performance comparison has proven to be a rigorous task, as the design of tools is algorithm-based, and motif models are manifold and complex. Numerous algorithms have been proposed, implemented and practiced in the last twenty years, which successfully generated a flood of sequence motifs by searching for the conserved pattern in functionally related genes. Usually, the choices made are not particularly stated, resulting in difficulties for the comparison of different implementations. Additionally, the implementation that has been employed is generally not comparable in a conclusive manner which the most promising ones.

Various methodologies defining a range of variation including both the underlying models together with algorithmic techniques have been proposed. Despite the significant effort of the researchers, the search for a potentially weak motif still stands as one challenging task, on the ground of the exponential time required for its execution or the large memory requirement. The volume of computationally as well as experimentally produced motifs and their increasing practicality make the motif search problem one of the outstanding components of computational biology.

## 6. Classification of DNA Motif Discovery

Several categories of the motif discovery process have been reported in the literature, from which primarily the following three variants are taken into account.

1. Simple Motifs Search (SMS);
2. Edited Motif Search (EMS);
3. Planted (*l*, d)-Motif Search (PMS).

Simple Motifs Search (SMS) [7]: These are the gene structure finding or predicting algorithms, used to identify the regions of DNA that encode RNA genes as well as the protein-coding genes and predict the regulatory regions. The simple motif search is the fundamental trend in sequence analysis operation. The input to the problem is a database (DB) of t sequences and an integer *l*, and the aim is to recognize all the length *l* motifs or patterns with their number of occurrences in the input sequences, with wild card characters entry arbitrarily present from 0 to $\left\lfloor \frac{l}{2}\right\rfloor$.

Edited Motif Search (EMS) [7]: These are the sequence alignment algorithms to align the biological sequences to figure out the similarity regions concerning the structural or functional or evolutionary relationship among the sequences. The alignment of amino acid or nucleotide is made in the same fashion as the rows of the matrix, and to align identical residues, in successive columns, gaps are inserted. The input to the problem is a database (DB) of t sequences and three integers *l*, d, and q. The aim is to identify the length *l* patterns occurring at least in the q input sequences.

Planted (*l*, d) Motif Search (PMS) [7]: These are treated as the most complex class of problems that search the biological sequence patterns capable of regulating the gene function. The problem takes t sequences, each of size n, and two integers *l* and d. The motive is to extract the binding site (planted variant) of motif M from each of the t sequences without any information of the planted variants’ locations. A variant of M is defined as a string of length-*l*, with at most d mismatch from M. (One variation to the planted (*l*, d) problem is the Extended (*l*, d) Motif Problem (EMP) [8] which finds the planted variant of motif M in at least k input sequences).

## 7. Planted (*l*, d) Motif Search and Its Representation

Mathematically, the planted (*l*, d) motif search is defined as follows: Given are n length, t sequences defined over a set of alphabet Σ and integers *l* and d with 0 ≤ d ≤ *l* ≤ n. The problem is to identify all length *l* substrings x which are present in every input string within Hamming distance d. Each of these x is referred to as an (*l*, d) motif.

For instance, there are three input strings *S*_1_ = TGTGTCA, *S*_2_ = GCGTATC, and *S*_3_ = GTTATGG. If the aim is to find out the motif of length 3 with Hamming distance at most 1 from every string, then TTA is a motif of interest, because the substrings “TCA”, “GTA”, and “TTA” of the strings *S*_1_, *S*_2_, *S*_3_, respectively, are the substrings with Hamming distance of at most one from TTA or are the variant (or instance) of the motif TTA.

There are three general methods of representing motifs: matrix representation, string representation, and regular grammar representation. In the matrix representation, motifs are expressed by the matrix model as a position-specific weight matrix (PSWM) or position weight matrices (PWMs) or position-specific scoring matrix (PSSM). The position weight matrix (PWM) representation uses a matrix consisting of nucleotide score, indexed by letter and position. Here, the observed nucleotide of a specific position in the motif is independent of the observed nucleotide of other positions ([9,10,11]). The positional weight matrix (PWM) can be generated by performing the column-wise normalization to effectively yield the sum of every column value as one. In the string representation, the motif is represented as the simple string of characters derived from the alphabet set ∑ = {A, G, C, T}, which is precisely defined by Pevzner and Sze [12]. In the regular grammar [13] representation models, it is assumed that a set of the rules can be obeyed by all the binding sites; however, for sequences of non-binding regions, it is not true. In this model, the extraction process of the optimal grammar out of a confined regular grammar class is exhaustive and leads to longer execution time.

### A Probabilistic Analysis of Motifs

A probabilistic analysis of motifs can be committed as follows. Assume *S*_k_ to be kth and the input sequence for 0 < k ≤ t and u to be an *l*-mer. Let the locations *l*, *l* + 1, 2*l* + 1, …, $\frac{n-l+1}{l}$*l* + 1 be starting positions. Each character can be viewed as an independent Bernoullian trial with the probability of success and failure as 3/4 and ¼, respectively, because the number of alphabets is 4 for DNA sequences. Then, the probability p for the occurrence of u in *S*_k_ by maximum d mismatches starting from an above-specified position is $\left(\begin{array}{c} l\\ d \end{array}\right){\left(\frac{1}{4}\right)}^{l-d}{\left(\frac{3}{4}\right)}^{d}$. We hit the motif if the given *l*-mer is present at most d letters mismatch. Then, we can calculate the probability of a hit $P_{\mathrm{hit}}$:

$$P_{\mathrm{hit}}=\overset{d}{\underset{i=0}{\sum }}\left(\begin{array}{c} l\\ d \end{array}\right){\left(\frac{1}{4}\right)}^{l-i}{\left(\frac{3}{4}\right)}^{i}$$

This probability does not take into consideration the overlaps *l*-mers. However, it gives an idea of the probability of a given pattern *S* with at most d mismatches at certain locations of a random sequence. Since in each sequence of length n there are at most (n − *l* + 1) possible *l*-mers, the probability that at least one of them will have up to d mismatches with the pattern P is ${\;P}_{1}=1-{\left(1-P_{\mathrm{hit}}\right)}^{\left(n-l+1\right)}$. Then, the probability that mutated pattern P is present in all t sequences is, $P_{t}=P_{1}^{t}$. Hence, the predicted numbers of (*l*, d) motifs are $E\left(l,d\right)\approx 4^{l}{\left(1-{\left(1-P_{\mathrm{hit}}\right)}^{n-l+1}\right)}^{t}$

## 8. PMS Algorithms

**Figure 1:**
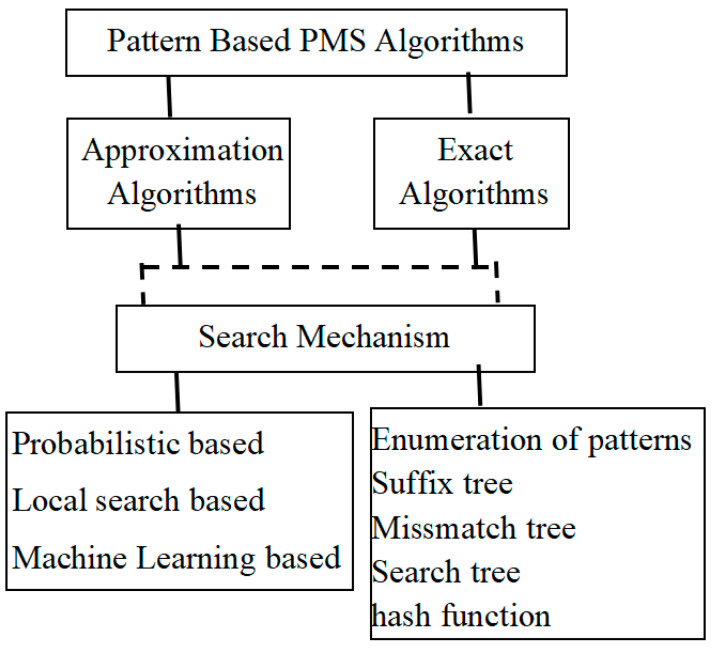
Hierarchy of PMS pattern driven algorithms.

Based on the fundamental approach of the PMS algorithm, two categories of motif search processes are available: one as profile-based approach and another as pattern-based approach. Profile-based approach determines the initial location of the motif occurrences in the input biological sequence, whereas the pattern-based approach determines the motif sequence itself. Owing to the importance of obtaining the required search pattern, the survey made in this paper is confined to only the pattern-based algorithms. The pattern-based approach initiates the process of local search with certain random candidate sequences ([14,15,16,17]). A pattern-based or profile-based approach can either be exact or approximate. The approximate approaches are generally heuristic in nature, and the guarantee of getting the optimal solution or correct solution is minimal. The exact algorithms run through a thoroughgoing search to ensure the correct solution. For the most part, PMS approximation algorithms are faster as well as popular compared to the exact algorithms; however, they are restricted to the scenario where approximate motif finding is sufficient. According to different researchers, the classification of planted (*l*, d) -motif discovery falls into the following categories: local search-based algorithms, probabilistic algorithms, genetic algorithm of Machine Learning-based approach, and the exact algorithms. Besides these other approaches that are also available in the literature, this paper attempts to analyze and review a few of the frequently found motif search algorithms under these categories. Figure 1 depicts the architectural view of the complete classification of the search algorithms according to the type of motif search as well as the search mechanism incorporated.

**A.**  **Probabilistic Algorithms**

Probabilistic algorithms for motif search mostly apply the statistical techniques of EM (Expectation Maximization) or Gibbs sampling or its extensions ([18,19,20]).

Expectation Maximization (EM) [20] carried out by E-step succeeded by the M-step. Initially, the E-step evaluates the expected likelihood of the perceived sequence data based on current settings of the argument, and then, the M-step updates these to optimize the expected likelihood function. The local optimization approach EM monotonously upgrades the expected likelihood; however, its sensitiveness to the initial position does not give any guarantee of convergence into a global optimum. Due to this drawback, the motif search algorithms which are based on EM proceed mostly from multiple initialization points to upgrade the likelihood of entering the global optimum. These heterogeneous initializations also enhance the chances of getting biologically suitable motifs that might not resemble the global optimum. Initially, the EM method was proposed for protein sequences, but later it became also applicable to DNA sequences. Assuming that every sequence consists of at least one common site, this model does not use any alignment of the sites. The EM algorithm handles the ambiguity of site position by applying the principle of missing information which allows recognition of the sites as well as the depiction of the binding motifs.

Gibbs Sampling [18] is an MCMC approach based on EM, and hence, it is applicable to the motif search problem with insufficient information. The uniqueness of the Gibbs sampling search procedure is undirected and global over a parameterized distribution from which it derives the random samples of hidden variants. The procedure is iterated by re-estimating the parameters based on the arbitrarily generated samples. But this global search of Gibbs sampling attracts a notable computational cost as it has to go through several iterations to converge to a computational likelihood surface. The assumption of a single occurrence of the motif for every sequence gives the method its name of “site sampler”. Like EM, in the Gibbs sampler, every step’s result depends on the previous step defining the “Markov Chain”, and the process of selecting the next step uses random sampling instead of being deterministic, defining “Monte Carlo” ([21,22,23,24]). The procedure extracts the length *l* substring from each of the t input sequences that maximize their “similarity”.

MEME [25] algorithm extends the EM algorithm to search the motif in a pool of unaligned biological sequences where insufficient prior knowledge about the motif is available. MEME initially gives input to the EM algorithm as the subsequence of the biopolymer sequence to accelerate the possibility of getting motifs that are globally optimum. It withdraws, the assumption of the presence of a single mutated motif per sequence. In order to discover the various distinct motifs in the same set of sequences, it incorporates a process of probabilistically eliminating the mutated motifs [19]. The algorithm receives a group of unaligned biological sequences and the motif length, and for each motif, it produces the motif model with its associated threshold value. This model is then treated as the optimal classifier for finding the occurrences of each motif in other datasets and returns an alignment of motif occurrences. In a single database, this process can be able to discover the distinctive motifs with their number of occurrences.

CONSENSUS [26] finds the functional relationship through the alignment of a group of related binomial sequences. For dissimilar types of aligned sequences, the score of relatedness is measured through a specific measurement strategy. Again, in the case of unknown alignment, it finds the alignment which optimizes the scoring scheme. The alignment is achieved by considering four components. First, the algorithm reviews the information content which is a log-likelihood scoring scheme. Second, it describes two methods for approximating the respective score of information content by a procedure that merges the technique of numerical calculations with the process of large-deviation statistics. Third, it describes the process of determining the number of probable alignments from the overall amount of sequence data. Fourth, it describes a greedy algorithm that identifies the alignment of functionally related sequences. Finally, it verifies the correctness of the calculation of the algorithm with an example.

Motif Sampler or Gibbs Sampler [27] enhances the robustness and performance of Gibbs sampling to noisy datasets. Noise in the data set is due to the presence of sequences not containing the motif or due to the large size of the input sequence as compared to the small size of the motif. The behavior of the algorithm in the presence of an increasing amount of noisy data has extensively been verified. The algorithm initially estimates the number of instances of the motif with the help of the probabilistic framework. Then, the author modifies the Gibbs sampling algorithm by introducing the higher-order background model (a transition matrix) based on an order m Markov process. All these modifications have been achieved in the iterative procedure of the Gibbs sampling.

BioProspector [28] builds upon the Expectation-Maximization (EM) method, addressing the problem of motif discovery for heterogeneous data. It combines two major characteristics of a motif’s consequences into one probabilistic score. The first one is the over representation which builds upon the number of occurrences of the motif in each input, and the second one is the cross-species conservation of individual motif occurrences over the input sequences. This approach enhances the algorithm of Expectation-Maximization to discover the motif for the given regulatory regions of co-regulated genes. It relates the species to any user-specified phylogenetic tree and evaluates the motif by relating its orthologous occurrences with a probabilistic model of evolution. The algorithm is also capable of managing the cases of incomplete heterogeneous data and thus makes it suitable for applications that handle incomplete orthology information.

MCEMDA [29] (Monte Carlo EM Motif Discovery Algorithm) is a Monte Carlo category of the Expectation-Maximization de novo motif search process that uses the technique of position weight matrix (PWM). It overcomes the drawback of traditional EM’s of being confined to a local optimum. In local alignment space, it carries out the stochastic sampling, which makes the popular Expectation-Maximization (EM) algorithm efficient. MCEMDA begins from one elementary model and successively applies the Monte Carlo simulation upgrading the parameters till the convergence. To manage the phase shifts and numerous modal issues, it introduces a log-likelihood profiling method. A novel GMA algorithm (Grouping Motif Alignment) forms the cluster or group of the population by locally aligning the candidates to select the strong motifs successfully. MCEMDA shows excellent capacity when compared to multiple sequence alignment methods. Table 1 summarizes the probabilistic algorithms.

**B.**  **Local Search Based Algorithms**

The algorithms that incorporate the local search approach might not be able to achieve the required planted motif always.

TEIRESIAS [13] is a combinatorial, local search algorithm, which is competent to output each pattern present in the minimum (user-defined) number of sequences. In the process of searching, it avoids the consideration of the entire search space successfully and hence is proved to be the efficient one. The algorithm uses regular grammar to represent motifs and report maximal patterns. It builds upon the approach that if an (*l*, w) pattern P is occurring in a minimum of k sequences, then its (*l*, w) sub-patterns also appear in at most k sequences, resulting from the largest patterns out of smaller sub-patterns. Through two phases, TEIRESIAS algorithm works scanning phase and convolution phase. The scanning phase applies the pruned exhaustive search to obtain all possible (*l*, w) patterns available in a minimum of k sequences having specifically *l* non-wildcards. In the convolution phase, these fundamental patterns are extended by gluing together. The quasi-linear running time of the algorithm with respect to the volume of generated output makes the algorithm output-sensitive in nature.

WINNOWER [12] is a graph-based algorithm, which finds the motif by searching a clique of the graph. It initially builds a set C of all feasible *l*-mers from each input sequence. Then, it constructs a graph where the node corresponds to the *l*-mer of set C, and edge corresponds to the connector of two *l*-mers from different strings if they are within 2d Hamming distance. In other words, for every motif, a corresponding clique of size t is available in the graph. This narrows the motif search problem into a clique finding problem of size t. This algorithm implicitly builds one t partite graph where each partition contains *l*-mers or nodes generated from t different sequences. Winnower treats all edges of the graph uniformly without making any distinction between the edges of high and low similarity, which requires considerable computational resources and is thus relatively slow.

SP-STAR [12], proposed by Pevzner and Sze, is a memory efficient, faster algorithm than WINNOWER. SP-STAR uses a sum-of-pairs scoring method D(W, *S*) = $\sum_{1<i<j<t}d\left(W_{i},W_{j}\right)$ to effectively make the distinction of signal patterns and random patterns and thus is separating signals from noise in a better way. This is a heuristic-based approach to find a pattern W that corresponds to min_W∈*S*_ D(W, *S*), using the same parameter as WINNOWER uses in its graph construction. SP-STAR score the *l*-mers along with the edges of G, accurately to eliminate more edges than Winnower does in its iteration.

cWINNOWER [30], proposed by Liang *S*, is the enhancement over WINNOWER [12]. Liang *S* improves performance by adding some consensus constraint on graph designing with an expectation of deleting the spurious edges in the initial stages. The consensus constraint contains stringent constraints to claim whether the edge under analysis should be a part of the t-clique or not. This algorithm identifies the fuzzy motifs of DNA sequences that are generous in protein-binding signals. Here, signal refers to a short (length *l*) nucleotide pattern with maximum d mutations from the motif. The algorithm searches those signals or motifs whose numerous mutated copies are found in the DNA sequences with sufficient abundance. The use of consensus constraint enables the detection of many weak signals.

In the Random Projection [14] algorithm proposed by Buhler and Tompa, the global search procedure is used to reach a better seed of the input which WINNOWER [17] failed to solve. It considers motif M as the *l*-mer and a set C as the group of *l*-mers from all given sequences. For some suitable value of k (where k < *l* − d and also k is not too small to operate with), it projects the *l*-mers of set C across the k randomly chosen positions, resulting in mapping from *l*-dimensional space into the k-dimensional subspaces. Then, it treats every k-mer as one integer and hashes them into the corresponding hashed group out of a total 4^k^ hashed group. If k < *l* − d, but it is not too small, then one hashed group can contain several *l*-mers. The highly enriched hashed group enables the recovering of motifs. It set the hashed group threshold to s for the elimination of the hashed group having *l*-mers less than s. By this process, the k-mers of the motif M and the hashed group become the same. For t sequences of length n each, the total expected *l*-mers are t(n − *l* + 1), and the possible number of k-mers is 4^k^. Thus, the expected *l*-mers per hashed group is t(n − *l* + 1)/4^k^, and the threshold value s is double its magnitude. Iteratively, the random hashing function h() is applied r times (for some appropriate r value), to ensure the hashed groups of size more than s are obtained at least once. Then, it collects all those *l*-mers for further processing to conclude with the final answer as motif M.

MULTIPROFILER [26] is a concept used by authors Uri Keich and Pavel A. PevZner, for searching the exact motifs in the DNA sequences. Multiprofiler generalizes the profile-based approach and enables to the identification of the exact consensus sequences that missed in the detection process of the standard profiles. This algorithm exceeds other major algorithms of motif search in many synthetic models.

Algorithm Pattern Branching [17] is built upon the local search techniques. For every l-mer of t input sequences, this procedure searches the neighbor *l*-mers and scores them properly. There are overall t(n − *l* + 1) *l*-mers available in t sequences, and against each *l*-mer, the total possible neighbors are $\left(\begin{array}{c} l\\ d \end{array}\right)3^{d}$, resulting in a sum of t(n − *l* + 1) $\left(\begin{array}{c} l\\ d \end{array}\right)3^{d}$ neighbors which is very large. As a solution, for a selected *l*-mers, the algorithm computes the best neighbor in an iterative fashion and outputs them as the motif.

Profile Branching [17] is a profile-based version of the pattern branching algorithm. It searches the motif in the motif profile space, instead of searching in the motif pattern space. It extends the pattern branching in several ways. It initially converts every sample string *S_i_* to a profile X(*S_i_*), and to score those profiles, it generalizes the scoring process. It also revises the branching method used in pattern branching such that it can be relevant to profiles and apply it iteratively k times (for suitable k value), on each *l*-mer of input sequences. Then, it executes the Expectation-Maximization algorithm to converge at a top-scoring profile. However, the pattern-based approach outperforms the profile-based approach by being five times faster on some challenging instances but fails to find the motif with numerous degenerate positions.

MotifCut [31] uses the graph-theoretic approach to address the problem of motif search. It initially constructs a graph considering the nodes as the substring edges as the connector of similar substrings. Then, it finds the maximum density sub-graph (MDS) in polynomial time. MotifCut exhibits two steps. First, it converts the given sequences to a group of K-mers, where the overlap and duplicate k-mers are considered as a distinct one and build a set of vertices. Second, it connects every pair of vertices through the edge, assigning one weight value. The weight value signifies the number of mismatches of the vertices. Then, it finds a higher degree of pairwise similarity between the vertices to construct the sub-graph representing the set of motifs. An important characteristic of MotifCut is that the approach is decidedly different from the frequently used PWM models, giving rise to significantly different motif generation. Table 2 summarizes motif discovery algorithms based on local search approach.

**C.**  **Machine Learning Based Algorithms**

In the literature, a collection of machine learning-based motif search approaches is available. Among these, the approach of genetic algorithm has a distinctive strength on the grounds of its frequent and popular usage [2]. The local search technique of standard machine learning does not reach the globally optimal solution. In spite of belonging to the family of the machine learning approach, the genetic algorithm flourishes due to its advantage in carrying out the global search to reach an acceptable global solution in genuinely less time.

FMGA [32] approach takes the benefit of the genetic algorithm strategy to output the better exact motif in less computation time compared to Gibbs Sampler [27] and MEME [25]. Initially, it computes and allocates a fitness score using a defined match function for each length *l* substring. Then, for every sequence, it sums up the individual fitness score to compute the value of Total Fitness Score (TFS). This step continues iteratively until the solution converges to an optimal state. In the process of convergence to the optimal solution, the next-generation candidate motifs are collected in two ways: one from the set of candidates with high TFS values and another through the selection process of the weighted wheel method. On the selected candidate motifs, it applies the process of the mutation using the position weight matrix to produce the parental patterns. Subsequently, to extract the new generation’s optimal child, it applies the crossover process on the parental patterns with uncertain code penalties. The essence of randomly generating patterns of FMGA justifies the solution for not reaching to the local minimum.

GAMOT [33] is one of the competent genetic algorithms to generate the optimal solution in minimum time for short as well as long motifs in a massive motif project. For the purpose of achieving the linear time and space complexity, GAMOT employs one additional motif finding step prior to the initiation of the evolutionary process of creating an extremely fit population. This contemporary motif finding step is the pivotal attribute of GAMOT that employs the total distance of the respective consensus as the scoring function. Taking the advantage of the scoring function, it deduces the highly occurring nucleotide from respective motif locations from the 4*^l^* feasible patterns. However, the similarity between the encountered consensus and the actual motif is very high, and owing to this reality, GAMOT minimizes the search space from 4*^l^* to (n − *l*)t consensus. As the initial step, GAMOT extracts the strings with good fitness and considers those to be the initial population. Then it applies iteratively the following steps to converge to the best and satisfactory individuals. It selects two distinct consensuses from the successive population through the process of linear ranking approach and creates next candidate consensus strings by employing a two-point crossover. In each iteration, it replaces the weak individuals with newly created individuals.

GAME [34] software uses the feature of the genetic algorithm framework for the extraction of the optimal motif. Unlike FMGA, GAME software takes advantage of the standard operators of the genetic algorithm used for motif search problem in addition to two extra operators SHIFT and ADJUST. The process of generation of the initial population happens through the random selection of the candidates which in turn minimizes the time and space of initial population generation. The simulation made by the software works with greater flexibility to focus only on the domain of the solution space among the entire population. The GAME software proves its dominance over BioProspector and BioProjector [28] and MEME [25] on various real data operations. The enhanced form of GAME performs better on manifold motif types.

GEMFA [35] is an EM-based genetic algorithm that integrates the heuristic search strategy of the genetic algorithm with the EM motif discovery method. This hybrid algorithm intelligently conducts the EM algorithm to easily escape from the locally minimal solution. One section of the GEMFA algorithm operates as a function optimizer by carrying out the multiple local arrangements as per the individual’s maximum likelihood and reducing the MDL (minimum distance length) value in every iteration of the genetic approach. The search process begins by taking a population into account through the multiple local alignments of encrypted chromosomes. Then, it moderately evokes the new population through the conventional process of reproduction by applying the steps of crossover, mutation, and selection until the optimal or best population is reached.

MOGAMOD [36] is one multi-objective genetic algorithm designed to locate the best motif existing in the given biological sequence. The backbone of MOGAMOD is the multi-objective algorithm NSGA-II [37] which is popular for its maximal performance. This multi-objective approach comprises a class of non-dominant initial population or solution. From these sets of the initial population, the algorithm extracts the best motif by revolutionizing the search process as a composition of three diverging optimization problems as maximization of similarity, enhancing the length of the motif and encouraging it to become the candidate motif. The performance of this algorithm is eventually improved by giving flexibility to the selection of a similarity measure in discovering the motif. The essence of the flexibility that exists in the selection process of the similarity index improves the performance of the MOGAMOD motif discovery algorithm.

GARPS [38] motif search process amalgamates the stochastic projection policy of random projection with the global search ability of genetic algorithms. The GARPS initially exerts the random projection policy on the input sequences by assimilating the position weight function and one original hash function h(s) formulated from the position weight function. This process generates the initial population of the worthy candidate with a dense signal for the iteration of the genetic algorithm. The random projection configures the k-mers from the respective *l*-mers by picking the consensus from randomly chosen k positions and designing a hash function h(s) from this. Based on the outcomes of the hash function h(s), the *l*-mers of individual sequences are hashed into the respective buckets. Then, from the enriched buckets of candidate motifs, the initial population is obtained. From the computed initial population, the refined candidate motif is obtained through the process of genetic algorithm iteration. The experimental result and the global search strategy of GARPS proves its efficiency and robustness as compared to the projection algorithm. Basically, GARPS is popular to handle the challenging instances and the weak motif in the group of the genetic algorithm approaches.

The algorithm AMDILM [39] proposes one revolutionary technique of motif discovering which exceeds the GARPS on the simulated sequences as well as on the real biological sequence. The algorithm begins with 64 distinct initial candidates of length three each. Then, iteratively, it imposes three operators of the genetic algorithm, mutation, addition, and deletion, in a sequence based on TFS values (Total Fitness Score) to increase the length of the motif to the desired one. For every candidate, the TFS value can be computed by finding the minimum distance of 64 candidates out of their corresponding maximum distances from the initial biological sequences. In every epoch of the iteration, the following three phases of operation take place employing the genetic algorithm operators until the length of the candidate motif reaches the length *l*. In the first phase, the mutation operator chooses one location from individual candidates and randomly replaces the location with any of the remaining nucleotides. In the second phase, the addition operator randomly inserts any nucleotide at the beginning and end of the candidate separately to have the two new candidates of length L+1 and preserves the candidate with more TFS value. Finally, in the third phase, the deletion operator removes the added nucleotide to carry forward the next iteration. To escape from falling into the local optimum, the AMDILM process starts the iteration from a distinct variety of initial populations.

GENMOTIF [40] (genetic algorithm to discover flexible Motif) is one genetic framework-based algorithm that uses the time series motif discovery method. It can be adjusted to any situation comfortably like probing in a span of segment length, associating with several dimensions, applying consistent scaling, and using multiple compatible grouping standards. It has two instinctive parameters, which once fixed within the limit never affect the performance of the algorithm. Due to this, GENMOTIF is named the parameter-friendly algorithm.

MHABBO [41] is one improved multi-objective biogeography-based optimization (BBO) algorithm that uses a differential evolution (DE) advance to discover a motif from DNA sequences and has gained excellent results. Its fitness function builds upon the information distribution within the habitat individuals. In each generation, the algorithm iteratively changes the migration probability and mutation probability based on the relationship among the average function cost and the fitness function cost. This algorithm redefines the fitness function based upon the information distribution and the Pareto dominance relation. The differential evolution (DE) concept combines the algorithm with the modified mutation procedure. It improves the migration operators that build upon the number of iterations to satisfy the requirement of motif discovery. Furthermore, immigration rates and emigration rates based on a cosine curve are modified to generate promising candidate solutions. The MHABBO algorithm performs better in terms of the quality of the final solutions.

KEGRU [42] model predicts the motif binding sites through Recurrent Neural Network (RNN) based on a convolutional neural network. It integrates a Bidirectional Gated Recurrent Unit network with K-mer embedding activity. In three phases, the model incorporates the process. First, it divides the DNA sequence into some specific strides and lengths and then names those as K-mers. Secondly, it uses the word representation algorithm “word2vec” by pre-training every K-mer to a corresponding word. Finally, it constructs a deep-learning-based GRU model for feature classification and feature learning. With experimental evidence, it validates the demand for being an inexpensive and timely motif extraction model.

DESSO [43] is a DL-based motif finding framework that performs in a better way than the existing tools beyond the state-of-the-art. It predicts cis-regulatory motif and TFBSs identification through binomial distribution and deep neural network. The performance of DESSO expands due to the integration of DNA shape feature detection. In addition to the prediction, DESSO can identify and analyze the structural binding sites through the integration of a deep-learning framework with DNA binding complexity. The experimental results demonstrate the insistence of identifying the mysterious or unknown motifs and their shape factors which were unidentified earlier.

The Hierarchical LSTM and attention network [44] method extracts the interdependency between various DNA, RNA, and protein sites. Instead of emphasizing motif discovery/prediction, it focuses on the region of the DNA or RNA binding sites through the application of the attention mechanism. On the ground of this, it is able to achieve the optimal combination of K-mer stride window, K-mer length, and its sentence length, and additionally, through hyper-parm experiment, it also reaches the optimization function. Table 3 summarizes motif discovery algorithms based on the machine learning approach.

**D.**  **Exact Algorithms**

The beauty of the PMS exact algorithm lies in the guarantee of furnishing all the real (*l*, d) motifs present in the given biological data. However, its NP-complete nature makes its worst-case execution time to be exponential in some parameters. For some specific type of problem, a scheme called Polynomial Time Approximation Scheme (PTAS) [45] is present, which can give polynomial-time execution in its worst case. Most of the known exact algorithm applies the process of search on a certain standard random sequence of data in the following fashion: Randomly, it creates twenty input sequences of size 600 each from the set of alphabets ∑ = {A, T, G, C}. Then, it creates a motif M of size *l* and inserts all given sequences with maximum d mutation to assure their existence in all the input sequences. Based on the values of *l* and d, explicitly, some occurrences of PMS have been recognized to be challenging. Any planted (*l*, d) occurrence is termed as the challenging instance if the number of predicted motifs occurring by random chance is one or more. For example, the challenging instances of motifs are (9, 2), (11, 3), (13, 4), (15,5), (17, 6), (19, 7), (21, 8), (23, 9), (25, 10), and (26, 11). In the literature, it is customary to compare the exact PMS algorithms performance specifically on these challenging instances.

In the way of obtaining a motif, the exact algorithms are categorized into two approaches: sample-driven and pattern-driven. The exact algorithm can use the concept of either pattern-driven or sample-driven or in some cases also a combination of both. From the given t sequences, the sample-driven approach enumerates all achievable (n − *l* + 1)t *l*-mers to compute the common neighborhood and outputs them as the motif. On the other hand, the pattern-driven approach enumerates all achievable ${\left|\sum \right|}^{\;l}$*l*-mers and outputs the *l*-mers that occur with a maximum frequency in all input sequences as the motif. The space complexity of the sample-driven approach is higher compared to the pattern-driven approach. The algorithm that uses the combination of approaches initially extracts the *l*-mers from multiple input sequences using the sample-driven approach and then generates the common d-neighborhood using the pattern-driven approach. Nonetheless, most of the biologists prefer the pattern-driven algorithm due to its lower space complexity.

The data structure or common method adopted by the exact algorithms are enumeration of patterns, search tree, suffix tree, tries, mismatch tree, linked list, graph, hash function, and so many. In the approach of pattern enumeration, from the input sequence, the feasible candidate motifs are enumerated and based on the expediency of being a motif, the search process is employed. The variation in this group of algorithms is based on the way of candidate motif enumeration. The group of algorithms in this category is PMS0 to PMS3 [46], PMSi and PMSP [47], Improved Pattern-driven [48], Stemming [49], PMS4 to PMS6 [50,51,52], PairMotif [53], etc. The approach based on the search tree constructs a depth d search tree of the candidate l-mer by position-wise changing its character in each iteration. Then, with the hope of getting the motif, it traverses in a depth-first order of the search tree by processing each node encountered in the traversal path. The group of algorithms falling under this approach are PMSprune [54], qPMSprune [55], Pampa [56], PMS3p [57], provable [58], and qPMS7 [55]. From the special tree-based approach (suffix tree, tries, and mismatch tree), the suffix tree is popularly used. The suffix tree approach applies some pre-processing on the input data to organize the data in a better way and to accelerate the process of motif search. It builds a single suffix tree from the input sequences by extending the motif length from zero to *l*. For every individual *l*-mer, a corresponding unique suffix tree can be built which produces a distinct depth order tree traversal sequence. From these sets of unique traversal sequences, the search process extracts the commonly found sequences and produces those as the motif. The group of algorithms from this category are SPELLER [59], SMILE [60], MITRA [61], CENSUS [62], PSMILE [63], RISO [64], RISOTTO [65], EXMOTIF [66], SLI-REST [67], etc. In this group, other than the suffix tree, MITRA [61], and CENSUS [62] uses the data structure mismatch tree and tries, respectively. The voting [68] algorithm uses the idea of the hashing search process. Some of the recent algorithms incorporate a combination of pattern-driven and sample-driven approaches to minimize the computational time. PMS8 [69] and qPMS9 [70] are the algorithms in this category.

PMS0 [46] algorithm receives t input sequences of n size each and constructs three groups of *l*-mers, namely, C, C′, C″ as follows. It teams up all possible t(n − *l* + 1) *l*-mers to group C, all (n − *l* + 1) *l*-mers of first sequence to group C′ and all $\left(\begin{array}{c} l\\ d \end{array}\right)\;\left(\left|\sum \right|-1\right)$ neighbors of each l-mer of set C′ to C″. Then, it evaluates the Hamming distance of *l*-mer u and *l*-mer v for u ∈ C and v ∈ C″ and produces the *l*-mer v as the motif those are available with maximum of d Hamming distance from every input sequence. For |∑| = 4 the time complexity of PMS0 is O(n^2^tl$\left(\begin{array}{c} l\\ d \end{array}\right)$3d).

PMS1 [46] algorithm takes the advantage of radix sort to do the sorting of a group of *l*-mers. It constructs the set Ci by bringing together all *l*-mers u of input sequence *S_i_* for 1≤ *i* ≤ t and the set Li by bringing together all neighbors v of *l*-mer u that are available maximum by d Hamming distances. The number of *l*-mers present in set Ci and *L_i_* is t(n − *l* + 1) and $O(n\;\left(\begin{array}{c} l\\ d \end{array}\right)\;{\left|\sum \right|}^{d})$, respectively. Then, using the process of radix sort, it sorts the *l*-mers of *L_i_* and hence removes the redundant neighbors. Then, it successively merges two consecutive sets, *L_i_* + 1 = *L_i_* ∪ *L_i_* + 1 for 1 ≤ *i* ≤ t, to figure out the common neighbors.

The improved pattern-driven [48] enhances the basic pattern-driven approach by giving the assurance of returning the optimal motif. Using the fundamental pattern-driven approach, it enumerates the possible 4*^l^* competent motifs with a computation time irrespective of the size of the given sequence, and hence, the complexity of the search is reduced by a factor of n. The search time of this algorithm is O(4*^l^l*t) for t input sequences. The process of search is accomplished in two steps. In the first step, on 4*^l^* competent motifs, it applies exhaustive search which simultaneously allows the invariant position and disallows the mismatches among the residues. In the second step, the attainable motifs go through the refinement process with a defined flexibility value to obtain the required motif. This algorithm can handle the motif with higher *l* and d values, compared to the basic one.

PMSi [47] improves the process of common neighborhood generation of PMS1 [41] by computing the pairwise relationship among the neighbors instead of handling at a time the whole mass of competent neighbors. For every single ith sequence (for 1 ≤ *i* ≤ t), it figures out the common neighbors of all the *l*-mers to a distinct group, namely, Li. Then, for every pair of sequences S_2*i*−1_ and S_2*i*_, it intersects its corresponding Li’s in a pairwise manner as $\bar{L_{i}}\;=\;L_{2i-1}\;\cap \;L_{2i}$ for *i* = 1 to t/2. At the end of the process, it intersects the entire lists of ${\bar{L_{i}}}^{’}s$ and generates the final motif set M. The pairwise approach of the common neighbor finding of PMSi substantially reduces the steps (computation time) and mass of the candidate motif (space requirement) of the original algorithm PMS1 [46].

PMSP [47] deploys the idea of the building neighborhood of every *l*-mers of the sequence S1, verifying the feasibility of being the (*l*, d) motif for each of these neighbors. To achieve this, it iteratively finds the set of neighborhoods let Z for every *l*-mer *x* ∈ S_1_, and for each *l*-mer *y* ∈ Z, it checks whether there exist *l*-mers in all the t sequences that are within Hamming distance d from it. Again in [54], the search process is reduced by the key observation that instead of checking the distance of *y* from every *l*-mers of all the sequences, it will be more appropriate to check only those *l*-mers that are at maximum 2d distance from *x*. PMSP uses O(tn^2^) space and takes O(tn^2^$\frac{1}{w}$N(*l*,d)) time, for a number of neighbors of any *l*-mer *x* as N(*l*, d). Although its worst-case time complexity is higher than the previous algorithms, it can solve challenging instances (15, 5) and (17, 6) in a reasonable time.

PMSprune [54] goes one step ahead of PMSP by including some original strategy. Analogous to the PMSP approach, PMSprune generates the neighbors for each l-mer *x* of the first sequence and then checks their feasibility of being a valid motif. However, the neighbor generation approach of PMSprune incorporates the efficient pruning technique due to which remarkable reduction in the search space happens. Initially, in a branch and bound method, it brings out the neighbors of l-mer *x* by constructing a tree T(*x*) of d height. Then, through the depth-first order traversal, it traverses the tree T(*x*), and for every *l*-mer *y*∈T(*x*), it computes the maximum of minimum distances ${\bar{d}}_{H}(y,S)$ by which y exists in each of the given sequences ‘S’. It outputs the set of y whose ${\bar{d}}_{H}(y,S)$ values are maximum d and prunes the nodes of the tree whose own ${\bar{d}}_{H}(y,S)$ value as well as the descendants ${\bar{d}}_{H}(y,S)$ values are higher than d. This state of the art of the pruning process makes this algorithm distinct from others and also exceptionally reduces space complexity.

Stemming [49] uses a novel process of neighborhood generation which reduces the computational search space by a factor of the size of the alphabet |∑|. The search process initiates with the neighborhood generation of the candidates followed by the intersection of the neighborhoods to form a set C with maximum m mismatches among themselves. This set C at the outset is considered to be the superset carrying a mix of motifs and some non-motifs. The set C can be characterized by the wildcards or stems where these wildcards or stems are expressed through motif length (*l*), Hamming distance (d) and maximum value of mismatch (m) only. As the stems are independent of the alphabet size, the computational search complexity is cut down to only the size of the candidate set C.

PMS4 [50] is a speedup method which can accelerate any PMS search process that is based on the following two-step practices: step one does the extraction of a group of candidate motifs from all given sequences, and then, step two verifies the candidate motifs against the characteristics of motif to obtain the true motif. The PMS4 speeds up any algorithm when it is fused with the algorithm. First, it does the extraction using the method of corresponding algorithm in k preferred sequences and labels it as the set C (this set C undoubtedly possesses all the actual (*l*, d) motifs with the non-motif candidates). Then, it verifies the validity of individual candidates of the set C for being the authentic (*l*, d) motif in time O(tn*l*). The selection of the value of k is the key feature causing the speedup, and its value differs from one fusion to another.

PMS5 [51] is the fusion of the PMS1 [46] and PMSprune [54] algorithms. It expands the idea of PMS1 by introducing the state of the art of neighbor generation of the PMSprune [54] algorithm. It receives a set of t input sequences and iteratively searches for (*l*, d) motifs from a triplet of sequence *S*_1_, *S*_2*i*_, *S*_2*i*+1_ for 1 ≤ *i* ≤ $\frac{t-1}{2}$. From the triplet sequence, it forms a group of 3 *l*-mers as (*x*, *y*, *z*) where $x\in_{l}S_{1}$, $y\in_{l}S_{2i}$, $z\in_{l}S_{2i+1}$, and using the concept of PMSprune, it works out the subroutine Bd(*x*, *y*, *z*) to explore the common neighborhoods of this *l*-mer group. The beauty of subroutine Bd(*x*, *y*, *z*) lies in the blending of the process of tree construction of PMSprune with the pre-processing step of Integer Linear Programming (ILP). The pre-processing step of ILP makes the process of extraction of common neighborhood faster compared to its competitor algorithms. However, the use of ILP intensifies the space requirement due to the use of a lookup table.

PMS6 [52] is the refinement of the algorithm PMS5. It skillfully reduces the searching time and space requirement by introducing improved pre-processing steps and one hashing technique for the lookup table, respectively. The distinctiveness of PMS6 compared to PMS5 lies in the way of thinking about the *l*-mer *x* of sequence *S*_1_ in the process of motif extraction. Here, the searching process is accomplished in two steps. In step one, it forms a triplet of *l*-mers (*x*, *y*, *z*) as of PMS5 and five equivalence classes C(n1, …, n5) according to the type of alliance of the nucleotides corresponding to the *l*-mer locations. Then, it places the triplet instance into its corresponding class according to the calculated value of n1 to n5. In step two, the set of motifs among *l*-mer $x\in S_{1}$, l-mer $y\in S_{2i}$, and l-mer $z\in S_{2i+11}$ for 1 ≤ *i* ≤ $\frac{t-1}{2}$ is determined by the principles of equivalence classes.

PairMotif [53] algorithm efficiently decreases the search space of the motif by introducing the state of the art of pairing concept. Initially, it pairs the candidate *l*-mers to different input sequences that differ with higher relative distances and then performs the extraction of motifs by iteratively performing the following three phases. In the first phase, it carefully selects the pair of *l*-mers which are at 2d Hamming distance by selecting one l-mer from sequence *S*_1_ and another from sequence *S*_r_ for 2 ≤ r ≤ t. The value of r is enumerated in a restrictive way. In the second phase, two filtering techniques are applied to the selected l-mer pairs to cut down the space of *l*-mers to be processed in the subsequent stages. This stage directs the candidate *l*-mers to the next phase; those are either the motif or tend to be the motif. Finally, the third phase computes the common neighbors among the filtrate candidates by applying the verification of being motif. The empirical outcome proofs its stableness and efficiency to handle a variety of length sequences compared to the existing algorithm of that period.

The algorithms which use a different data structure, such as the suffix tree, or try to or mismatch the tree are described as follows.

Speller [59] is the first algorithm to introduce the data structure suffix tree to speed up the motif search process. From the given input sequence, it builds a suffix tree in a lexicographic manner that yields unique patterns when is traversed from the root node to any leaf node. On the suffix tree, it applies some pre-processing to handle the gap among the candidate motifs. Then the selection of motif from the suffix tree is done in two steps: step one pulls out the duplicate motif, and step two draws out the common motif. Step one determines the availability of common patterns with some allowable distances in only q (quorum constraint) sequences from the group of t sequences. Step two determines the motif by verifying the feasibility of the generic motifs to become the motifs. The space complexity of SPELLER is O(nt^2^/w) with time requirement O(nt^2^N(*l*, d)) where w is the length of a word, and N(*l*, d) stands for the number of neighbors that any *l*-mer can have. The introduction of the suffix tree makes easy to organize and pre-process the input data.

The SMILE [60] algorithm represents all suffixes of the given sequences through the generalized suffix tree in contrast to the classical suffix tree. A distinctive feature of a generalized suffix tree is to assign the unique termination symbol for an individual input sequence and use a bit vector per node to specify the sequence name described by the path from root to leaf. Utilizing the DFS recursive traversal process, the algorithm traverses the suffix tree T by considering it to be a classical lexicographic suffix tree, intending to depict every feasible length l motif. Similar to the SPELLER, it applies the initial search phase on quorum q of input sequences. The algorithm performs the bitwise OR operation on the bit vectors of the nodes that are encountered in the path of motif search, i.e., the set of nodes encountered from the root to that node, and depending on the presence of ones in the bit vectors, it returns the motif. In every step of depth-first traversal, it prunes the set of nodes that lack the required features or lie below the threshold value and backtracks to survey the new valid path.

Algorithm MITRA [61] exploits the pairwise resemblance of *l*-mers by a combinational approach of WINNOWER with the theory of the suffix tree-based approach of SMILE. It constructs a depth *l* mismatch tree where the degree of each internal node is |∑|, described by the unique value from ∑. The search space of the motif is split into subspaces at each level, starting from root to that node corresponding to its unique prefix code. These subspaces are described by the path label and keep track of the *l*-mers that is found to be present by maximum d mismatches. In the process of the traversal, the algorithm spells out the availability of the patterns P with d Hamming distances in at least the quorum input sequences or the corresponding input subspaces. In the search process, if any subspaces are predicted to be weak, then those subspaces starting from the root are pruned and backtracked to the next path, and if successful, the subspaces are further explored to be processed down. As the pruning technique MITRA only allows the valid subspaces concerning to the motif, the depth l mismatch tree at the end reports the valid motif only.

CENSUS [62] parallel algorithm gives a solution to the space component of the generalized suffix tree by eliminating the node wise bit vector storage concept. It constructs a t number of tries or a lexicographic tree for t input sequences by briefly encoding the corresponding *l*-mers in time O(lnt). Then, in accordance with the reality that multiple motifs can share a common prefix, this algorithm explores iteratively the potential motif for only the possible prefixes of the motif to avoid the unnecessary processing. The encoding process of the tries looks at the count of the availability of motif instead of the size of the motif. Owing to the one-to-one correspondence between the tries and the input sequence, CENSUS uses a distributed memory parallelization technique to eliminate any sharing of information.

PSMILE [63], the parallel SMILE algorithm, efficiently extracts the structured motif through the use of the fundamental suffix tree. PSMILE parallelizes the technique of SMILE by the introduction of the P number of same-sized buckets with some allowable errors and interval of distances in the consecutive buckets. As at the initial stage of the algorithm, the motif or the content of bucket is untold, the process represents the bucket space through tries or lexicographic tree. However, for some exceptional instances, the algorithm uses the suffix tree of given sequences. The optimality of PSMILE is achieved through an additional balancing technique, i.e., the process of balancing the motif extraction over the existing processor. In this balance partitioning approach, the search space is equivalently split and distributed among the individual loosely coupled processing units, and thus, the speed up is achieved proportional to the number of processing units.

RISO [64] algorithm extends the SMILE algorithm with a twofold process. In the first step, a suffix tree called the factor tree is built up to a certain level *l* instead of considering the complete suffix tree of the entire input sequences. This step specifically reduces the storage requirement of the SMILE. Then, in the second step, it proposes a data structure named a bucket link, which caches the situations required to pass from one bucket to another through the link. The process winds up by thoroughly extracting the subsequent motifs by partially or temporarily modifying the factor tree. Contrary to SMILE, it saves time and reduces search space by not using the pruning technique in the second step.

RISOTTO [65] based on the idea of PMS0 upgrades the proficiency of RISO by tying up the box-link data structure with the suffix tree data structure. For the given sequence S_1_, it builds suffix trees describing the d-neighborhood for every *l*-mer and explores it in a depth-first way. In the search process, it steps aside the exploration of the new node for which quorum is satisfied or ultimate length is reached. RISOTTO minimizes the computation time of RISO by avoiding the conflicting type of candidates through the mechanism of caching the maximum extensibility factor information. However, the supplementary space is required for the extensible information storage.

EXMOTIF [66] is the well-known structured motif algorithm which outperforms the RISO algorithm [64], in approximate matching as well as exact matching. This algorithm also surpasses the RISOTTO algorithm [65] by presenting the real occurrence of the structured motif rather than presenting the relative number of occurrences as was suggested by RISOTTO. It operates with a variant of the data structure suffix tree consisting of the inverted index of symbol locations. This contributes to enumerating the structured motif through positional joins over the index.

SLI-REST [67] (Suffix Link on Internal nodes, a Reverse Engineering Suffix Tree) applies the reverse engineering method on the suffix tree and links. It takes a tree of all input sequences, performs some modification to it, and searches for a word whose suffix tree is isomorphic to the input tree. It constructs the suffix tree in linear time by incorporating multiple suffix links or edges to all the internal nodes and investigates the suffix tree using the reverse engineering process through one novel approach. Before employing this novel approach, the author first presents the required constraint for a candidate tree and then defines a bicolored directed graph with proper labelling on its edges for every internal node which is the direct parent of the leaf nodes. Lastly, in the graph, the algorithm explores the particular feasible Eulerian routes, for which the traversed edge labels meet the required conditions and outputs a word achieving the given suffix tree and links.

PAMPA [56], a branch and bound algorithm, enhances the PMSprune [54] by efficiently managing the search space of the motif. The theoretical time and space requirements are the same as PMSprune, but in the practical aspect, it scales down to half for the challenging patterns. It describes Bd(*x*), the d-neighborhood of an *l*-mer *x*, by introducing the extended *l*-mers with “wildcards” to represent any symbol from the set of alphabets. Thus, the concept of “distance” ${\bar{d}}_{H}(.,S)$ of the PMSprune is refined in PAMPA for extended *l*-mers in a precise way and is used in the evaluation of ${\bar{d}}_{H}(.,S)$ for the d-neighborhood Bd(*x*).

PMS3P [57] efficiently integrates the idea of PMS3 with the feature of PMSprune to handle the challenging motif instances. Making use of splitting concept of PMS3, it splits the *l*-mers of sequence one into two-part u and v of size *l*1 and *l*2, respectively. Then utilizing the tree construction and pruning concept of PMSprune it constructs tree T(u) of height h for *l*1-mer u and tree T(v) of height d-h for *l*2-mer v. Then, individually, it explores both the trees T(u) and T(v) in a DFS way by applying the pruning technique. In the process of pruning of trees T(u) and T(v), it extracts the neighbors which are at Hamming distance h and d-h, respectively, from all input sequences, and saves them in groups Q′ and Q″, respectively. Then, the corresponding groups Q′ and Q″ of every *i*th input sequence are merged to attain a list Ai in Appendix A, and finally, all Ai lists are intersected to return the final planted motif. Though the computation time exceeds the previous algorithm like PMSP and PMSprune, the proficiency to handle the challenging instances is very high.

The provable [58] algorithm employs the extended closest string (ECS) problem in place of the simple closest string problem in a recursive manner to extract the planted motif. Taking advantage of the two closest string algorithms, it extracts the center string from the group of input strings with some acceptable substitution. The author has proved its correctness by experimenting with some challenging instances on the real data set. The beauty of the algorithm lies in its extension version named center substring algorithm, which is powerful enough to explore all types of solutions to the problem. For every given sequence, the extended algorithm can find all possible center substrings and can transform into the fast exact algorithm of planted motif search with some modification.

qPMSprune [55] is the first efficient exact algorithm to solve the planted quorum (*l*, d, q) motif for the challenging instances with larger *l* and d values. A planted quorum (*l*, d, q) motif problem searches the length *l* planted motifs which occur in a minimum of the q input sequences, with at most d Hamming distance. Among the t input sequences, the qPMSprune finds one particular sequence S*_i_*, where 1 ≤ *i* ≤ (t − q + 1) and an *l*-mer u of Si such that one of its neighbors is M and M is an (*l*, d, q − 1) planted quorum motif of the given input sequences excluding Si. The algorithm processes every *l*-mer of Bd(u) to identify the (*l*, d, q − 1) motifs exploiting the pruning method of PMSprune [54].

qPMS7 [55] is a robust algorithm in the group of quorum planted motif discovery algorithms. It realizes and extends the idea of qPMSprune as follows: for the existence of two sequences S*_i_* and *S*_j_ for 1 ≤ *i* ≠ j ≤ t if *l*-mer u and *l*-mer v of sequences S*_i_* and *S*_j_, respectively, M can be (*l*, d, q − 2) planted quorum motif contingent upon M ∈ {Bd(u) ∩ Bd(v)}. Hence as an extension, it scrutinizes over such (i, j) sequence pairs by constructing one cyclic graph Gd(u, v) of *l*-mer u and *l*-mer v. Then, it explores the Gd(u, v) graph in a depth-first way to recognize all components of Bd(u) ∩ Bd(v), and those can be (*l*, d, q − 2) planted motifs.

Voting [68] algorithm is a two-step algorithm: one is simple voting, and the other is an improved projected voting algorithm. The observation of a simple voting procedure states that for motif M and its d-neighborhoods N(M, d), if mi is a neighbor of M, then definitely M is a member in the group of N(mi, d). Due to this observation, it gives a thought to the search of motif that any *l*-mer u present in N(M, d) should award one vote to other sequences *l*-mer, and the *l*-mer which receives a vote from all input sequences should be declared as the motif. The hash table manages and records the received votes of each *l*-mer, and through the tracking process, it outputs the *l*-mer having votes from every sequence as the motif. However, the computation time and storage requirement of the simple voting algorithm increase proportionate to the growth of *l* and d values. To overcome this demerit, the author extends the simple voting to the improved projected voting algorithm by minimizing the set of *l*-mers through the process of random projection. Then the author improves further by only considering the selected positions that are competent to the previously attempted positions.

PMS8 [69] incorporates the novel idea of neighbor generation to introduce the exact and efficient solution to the planted (*l*, d) motif search. It reveals the necessary and sufficient relation among three l-mers to get hold of common neighborhoods. The algorithm executes two steps: first, it applies the sample-driven approach to generate all sets of l-mers, and then, it applies the pattern-driven approach to extract the patterns present in all sequences. In the sample-driven step, it builds a matrix R of size t(n − l + 1) consisting of all l-mers of all sequences such that the *i*th row of matrix R corresponds to all l-mers of ith sequences. Then, it randomly chooses one l-mer u from the first row, pushes it to the stack and pulls out all l-mers v from the matrix R which are at Hamming distance higher than 2d from u. It repeats this process for each row with one twist, i.e., it removes the l-mer v not present in the group of common neighbors. The transition from the sample-driven step to the pattern-driven step happens when the size of the stack attains the threshold value. In the pattern-driven step, it generates the common neighborhood of l-mers of the stack and inspects the existence of any planted (*l*, d) motif in that group.

qPMS9 [70] is the most efficient and recent quorum parallel planted motif search algorithm that can improve efficiently the computation time of PMS8 [69]. In several ways, it extends PMS8, first, by introducing the state of art of string reordering mechanism which conspicuously improves the performance by employing the novel pruning method on the motif search space. Additionally, it assists the qPMS challenging instances that were missed in PMS8. The instances (28, 12) and (30, 13) are first solved by this technique with a reasonable time in a single-core machine. Table 4 summarizes motif discovery algorithms based on exact motif finding approach.

## 9. Conclusions

This article concisely discusses some of the planted (*l*, d) motif search processes with their comparison studies. The observation states that the success rate of computational motif search is higher in simple organisms such as yeast as compared to the higher organisms with more complex genomes. The progress towards achieving the approximate as well as the exact motif is very encouraging in the present era. Several methods representing a large variation concerning both the underlying model as well as algorithmic approaches have been proposed. However, the requirement to have one exclusive method which can consider all relevant aspects is the main concern. Keeping this in mind, different researchers proposed and implemented various motif search algorithms and models over a decade. After all, in different circumstances, the complex and diverse features of a motif are exposed as a stumbling block in the comparison of different motif discovery approaches and create obstacles in the course of best motif identification. It is observed from the research papers that even though the exact algorithm takes exponential time in its worst case, it does not necessarily mean that it will never solve the practical instances within a reasonable amount of time. In some cases, the approximate algorithm is acceptable, but the exact algorithms are found to be preferable to the biologists due to their capacity to report all the (*l*, d) motifs.

## Figures and Tables

**Table 1:** Summary of Motif Discovery Algorithms based on Probabilistic Approach.

S/N	Algorithm(s)	Commonness	Conclusion
1	Expectation Maximization (EM) and BioProspector	Both algorithms are based on local optimization approach or can find the motif which is confined to a local optimum	BioProspector is better due to its applicableness to heterogeneous sequences
2	Gibbs Sampling and Motif Sampler	Both use Markov Chain Monte Carlo approach and are time consuming	Motif Sampler is more robust and applicable to noisy datasets
3	MEME and CONSENSUS	Both the algorithms align a group of related binomial sequences to extract the common sequence motif	MEME consumes more space than CONSENSUS. CONSENSUS is Applicable to unknown alignment.
4	MCEMDA	This algorithm uses position weight matrix (PWM) and is the best in probabilistic approach	Globally optimum

**Table 2:** Summary of Motif Discovery Algorithms based on Local Search Approach.

S/N	Algorithm(s)	Commonness	Conclusion
1	WINNOWER, cWINNOWER, MotifCut	All are graph-based algorithms	cWINNOWER is relatively fast and able to identifies the fuzzy motifs
2	MULTIPROFILER, Pattern Branching and Profile Branching	Based on profile-based local search Techniques	Efficiency in finding the motif with numerous degenerate positions increases from MULTIPROFILER to Profile Branching
3	SP-STAR and Random Projection	Uses Sum-of-pairs scoring method which is the lobal search procedure	Memory efficient, faster algorithm and can reach the better seed
4	TEIRESIAS	Uses regular grammar to output each pattern that is present in the minimum number of sequences	Quasi-linear running time and output sensitive

**Table 3:** Summary of Motif Discovery Algorithms based on Machine Learning Approach.

S/N	Algorithm(s)	Commonness	Conclusion
1	FMGA, GAME	All are based on Position Weight Matrix and Random Selection	GAME is better than FMGA but more complex in its implementation.
2	GEMFA, MOGAMOD, and MHABBO	All are genetic based algorithm approach	All have the common stumbling block of using more search space. MHABBO is more efficient in this group.
3	KEGRU and DESSO	Both are based on neural network. It can predict with shape feature detection of unknown motif in less time and cost	For some fixed K value, the prediction is more appropriate
4	GAMOT, GARPS, and AMDILM	Based on Random Projection Strategy where it uses total distance as scoring function	AMDILM is more efficient in this group as it works with consistency and high accuracy
5	GENMOTIF and Hierarchical LSTM	These are time series motif discovery algorithms. Uses more search space.	Flexible enough to accommodate task characteristics and all type of motif specification and reach to achieve optimization Function

**Table 4:** Summary of Motif Discovery Algorithms based on Exact Approach.

S/N	Algorithm(s)	Commonness	Conclusion
Based on Enumeration of Patterns			
1	PMS0, PMS1, PMSi, PMSP	Algorithms of Rajsekaran and his groups which are based on building neighborhoods of a group of *l*-mers	Implementation is easy to compare with other methods but takes more search space
2	Improved Pattern-driven, Pair-Motif and Stemming	Novel process of neighborhood generation through the pairing concept. Pair-Motif is the best algorithm in this group.	Efficiently decreases the search space of the previous group. Stable and efficient to handle a variety length sequences
3	PMS5 and PMS6	Advance version of the first group of algorithms which includes hashing and ILP techniques	Use of ILP intensifies the space but gives accurate result
4	PMS4	Speedup Method which can actuate any motif search algorithm	Not self-sufficient. Needs a supportive algorithm to run
Based on Search Tree Approach			
5	PMSprune, qPMSprune, PMS3p and qPMS7	Incorporate the efficient pruning technique into the search tree of input (quorum) sequence	Efficiently handle the challenging instances with remarkable reduction in the search space. qPMS7 is the robust algorithm in this group
6	PAMPA	A branch and bound algorithm	Efficiently managing the search space of the motif
7	Provable	Employs the extended closest string	Solve the challenging instances on the real data set
8	PMS8 and qPMS9	Based on state of the art of string reordering mechanism and novel pruning method	PMS8 is efficient for smaller sequence. qPMS9 is most efficient and recent quorum parallel
Based of Different Tree Data Structure and Hashing Search Process			
9	SPELLER, SMILE, PSMILE, RISO, RISOTTO, SLI-REST and EXMOTIF	All are based on a suffix tree data structure	Save time and reduce search space. In this group, EXMOTIF is the finest to carry out approximate as well as exact matching
10	MITRA	Uses a mismatch tree and the pruning technique for minimizing the space	Combinatorial approach of Winnower and Smile
11	CENSUS	Uses tries to give a solution to the space component of the generalized suffix tree	Eliminates any sharing of information
12	VOTING	Uses easy tracking process of votes through hashing search process	Computation time and storage requirement are higher

## Data Availability

Not Applicable.

## Appendix A

**Table A1:** List of Motif Discovery Algorithms based on Probabilistic Approach.

S/N	Algorithm	Operating Principle	Strengths	Weakness	Author and Reference
1	Expectation Maximization (EM)	Local optimization approach	Chances of getting biologically suitable motifs are higher	Sensitive to the initial position and do not give any guarantee to converge into a global optimum	Lawrence et al. [20]
2	Gibbs Sampling	MCMC approach	Global over a parameterized distribution	Suffers a notable computational cost	Lawrence et al. [18]
3	MEME	Alignment of motif	Applicable to unaligned biological sequences with insufficient prior knowledge	More space required	Bailey et al. [25]
4	CONSENSUS	Alignment of a group of related binomial sequences	Applicable to unknown alignment	Uses Greedy algorithm	Hertz et al. [26]
5	Motif Sampler or Gibbs Sampler	Iterative procedure of the Gibbs sampling	Robust and applicable to noisy datasets	Time consuming	Thijs, G. et al. [27]
6	BioProspector	Expectation-Maximization (EM) method	Applicable to heterogeneous data	Confined to a local optimum	Sinha et al. [28]
7	MCEMDA	Position weight matrix (PWM)	Globally optimum	-	Bi CP. et al. [29]

**Table A2:** List of Motif Discovery Algorithms based on Local Search Approach.

S/N	Algorithm	Operating Principle	Strengths	Weakness	Author and Reference
1	TEIRESIAS	Uses regular grammar	Output each pattern that present in the minimum number of sequences	Quasi-linear running time and output sensitive	Rigoutsos et al. [13]
2	WINNOWER	Graph-based algorithm	Convert motif search to a clique finding problem	Requires considerable computational resources and is thus relatively slow	Pevzner et al. [12]
3	SP-STAR	Sum-of-pairs scoring method	Memory efficient, faster algorithm	Heuristic-based approach	Pevzner et al. [12]
4	cWINNOWER	Uses consensus constraint on graph designing	Identifies the fuzzy motifs	More space requirement	Liang et al. [30]
5	Random Projection	Global search procedure	Reach the better seed	-	Buhler et al. [14]
6	MULTIPROFILER	Profile-based approach	Better for the synthetic models	-	Keich et al. [26]
7	Pattern Branching	Local search techniques	Faster than profile-based approach	Fails to find the motif with numerous degenerate positions	Price et al. [17]
8	Profile Branching	Extends the pattern branching	Able to find the motif with numerous degenerate positions	Slower	Price et al. [17]
9	MotifCut	Graph-theoretic approach	Different from the frequently used PWM	-	Fratkin et al. [31]

**Table A3:** List of Motif Discovery Algorithms based on Evolutionary Approach.

S/N	Algorithm	Operating Principle	Strengths	Weakness	Author and Reference
1	FMGA	Position Weight Matrix and weighted wheel method	Gives solution for not reaching the local minimum.	Uses random generation-based prediction	Falcon et al. [32]
2	GAME	Position Weight Matrix and Random Selection	Eliminates the reliance on additional motif-finding programs	More complex	Wei et al. [34]
3	GEMFA	EM based genetic algorithm	Escapes from the locally minimal solution	Uses more search space due to heuristic search	Chengpeng et al. [35]
4	GAMOT	Uses total distance as scoring function	Fast Motif Discovery with smaller search space	Not tested on real data set.	Pevzner et al. [33]
5	MOGAMOD	Multi objective genetic approach	Flexibility exists in the selection process	Can only work with data set with sequential character	Kaya et al. [36]
6	GARPS	Random Projection Strategy	Optimize the heuristic method.	Process fails for the skewed nucleotide distribution	Hongwei Huo et al. [38]
7	AMDILM	Iteratively process the increased length motifs	Works with consistency and high accuracy.	Time consuming in finding fitness score iteratively	Yetian Fan et al. [39]
8	GENMOTIF	Time series motif discovery	Flexible enough to accommodate task characteristics and all type of motif specification	More search space required	Joan Serra et al. [66]
9	MHABBO	Differential evolution and multi-objective optimization	Diversity in population is maintained	Algorithm has not applied to multi-objective motif discovery problem	Siling Feng et. al. [67]
10	KEGRU	Recurrent Neural Network (RNN)	Inexpensive and Fast	Prediction done for some fixed ‘K’ values	Shen Z et. al. [68]
11	DESSO	Binomial distribution and deep neural network	Prediction with shape feature detection of unknown motif	Time Complexity	Yang J et. al. [69]
12	Hierarchical LSTM and attention network	Hierarchical LSTM and attention mechanism	Reach to achieve optimization Function	-	Shen Z et. al. [70]

**Table A4:** List of Exact Motif Discovery Algorithms based Enumeration of Patterns.

S/N	Algorithm	Operating Principle	Strengths	Weakness	Author and Reference
1	PMS0	Comparison of neighbor distances	Easy to implement and understand	More Space complexity and time complexity	Rajasekaran et al. [41]
2	PMS1	Radix Sort	Easy to implement and track the neighbors	More Space complexity and time complexity	Rajasekaran et al. [41]
3	PMSi	Compute the pairwise relationship among the neighbors	Pairwise approach reduce computational time and space	Computes the common neighbors of all *l*-mers	Davila et al. [42]
4	PMSP	Based on building neighborhood of all *l*-mers of first sequence	Can solve previously unsolvable instances	Space consuming	Davila et al. [42]
5	Improved Pattern-driven	Fundamental pattern-driven approach	Handles the motif with higher l and d values	More time complexity	Sze et al. [43]
6	Stemming	Novel process of neighborhood generation	Reduces the computational search space	Not able to reduce time of computation	Kuksa et al. [44]
7	PMS4	Speedup method	Actuates any motif search algorithm	Not self-sufficient. Needs a supportive algorithm to run	Rajasekaran et al. [45]
8	PMS5	Fusion of PMS1 and PMSprune. Use ILP in preprocessing step	More time complexity	Use of ILP intensifies the space	Dinh et al. [46]
9	PMS6	Introduces improved pre-processing steps and hashing technique	Skillfully reduces the searching time and space requirement	Higher number of equivalence classes are used	Bandyopadhyay et al. [47]
10	Pair-Motif	Introduces the state of the art of pairing concept	Efficiently decreases the search space. Stable and efficient to handle a variety length sequences	-	Yu, Q. et al. [48]

**Table A5:** List of Exact Motif Discovery Algorithms based Search Tree Approach.

S/N	Algorithm	Operating Principle	Strengths	Weakness	Author and Reference
1	PMSprune	Incorporates the efficient pruning technique on the search tree	Remarkable reduction in the search space	After the tree construction, pruning happens not at the time of tree construction	Davila et al. [49]
2	qPMSprune	Search is applied on quorum of input sequences	First efficient exact quorum algorithm to solve challenging instances	-	Dinh et al. [50]
3	PAMPA	Branch and bound Algorithm	Efficiently managing the search space of the motif	Theoretical time and space requirements are the same as PMSprune	Davila et al. [51]
4	PMS3p	Efficiently integrates the idea of PMS3 with the feature of PMSprune	Proficiency to handle the challenging instances is very high	Computation time exceeds from some of the previous algorithms	Sharma et al. [52]
5	Provable	Employs the extended closest string	Solve the challenging instances on the real data set	-	Chen et al. [53]
6	qPMS7	Extends the idea of qPMSprune	Robust algorithm in the group of quorum	More space requirements	Dinh et al. [50]

**Table A6:** List of Exact Motif Discovery Algorithms based on Suffix tree, Mismatch tree, Tries, and Hashing Search Process.

S/N	Algorithm	Operating Principle	Strengths	Weakness	Author and Reference
1	SPELLER	Suffix Tree	Speeds up the motif search process	Extra pre-processing is required	Sagot et al. [54]
2	SMILE	Suffix Tree	Uses generalized suffix tree in contrast to the classical suffix tree	More space required due to the use of node-wise bit vector	Marsan et al. [55]
3	PSMILE	Suffix Tree	Efficiently extracts the structured motif	For some exceptional instances, it does not work properly.	Carvalho et al. [58]
4	RISO	Suffix Tree	Saves time and reduces search space	-	Carvalho et al. [59]
5	RISOTTO	Box-link data structure with suffix tree	Upgrades the proficiency of RISO	Supplementary space is required	Pisanti et al. [60]
6	EXMOTIF	Suffix Tree	In approximate matching as well as exact matching, it out-performs Riso	-	Zhang et al. [61]
7	SLI-REST	Reverse engineering method on the suffix tree and links	Fast response	Explores the feasible Eulerian routes	Cazaux et al. [62]
8	MITRA	Mismatch tree	Uses pruning technique to minimize the space	Combinatorial approach of Winnower and Smile	Eskin et al. [56]
9	CENSUS	Tries	Gives a solution to the space component of the generalized suffix tree	Eliminates any sharing of information	Evans et al. [57]
10	VOTING	Hashing search process	Uses easy tracking process of votes	Computation time and storage requirement is higher in simple voting	Chin et al. [63]
